# Knowledge, attitudes and perceptions towards polio immunization among residents of two highly affected regions of Pakistan

**DOI:** 10.1186/s12889-015-2471-1

**Published:** 2015-11-05

**Authors:** Muhammad Umair Khan, Akram Ahmad, Talieha Aqeel, Saad Salman, Qamer Ibrahim, Jawaria Idrees, Muhammad Ubaid Khan

**Affiliations:** Department of Clinical Pharmacy, UCSI University, Kuala Lumpur, Malaysia; Department of Pharmacy Practice, Faculty of Pharmacy, University of Balochistan, Quetta, Pakistan; Department of Pharmacy, University of Peshawar, Peshawar, Pakistan; Sindh Government Hospital, New Karachi, Karachi, Pakistan

## Abstract

**Background:**

Despite the efforts of national and international organizations, polio has not been eradicated from Pakistan. The prevalence of polio in Pakistan is exceptional in global context. Quetta and Peshawar divisions are amongst the most affected regions hit by polio in Pakistan. This study was carried out to assess the knowledge, attitudes and perceptions towards polio immunization among residents of Quetta and Peshawar divisions in Pakistan.

**Methods:**

A descriptive, cross-sectional study involving 768 participants was conducted from August to December, 2014 in Quetta and Peshawar divisions in Pakistan. Multistage sampling technique was used to draw a sample of residents from each division. A pre-tested, self-administered questionnaire was used to collect the data from eligible participants. Descriptive and logistic regression analyses were used to express the results.

**Results:**

A total of 38.8 % participants exhibited good knowledge about polio. Mean knowledge score of the participants was 7.35 ± 2.54 (based on 15 knowledge questions). Older age (*p* < 0.001), low qualification (*p* < 0.05), rural locality (*p* < 0.05) and Quetta division (*p* < 0.001) were significantly associated with poor knowledge of polio. A large proportion of participants displayed negative attitudes towards polio immunization (84.8 %), with a mean score of 19.19 ± 2.39 (based on 8 attitude statements). Lack of education (*p* < 0.001) and rural residence (*p* < 0.001) were significantly associated with the negative attitudes of participants towards polio immunization. False religious beliefs (39.06 %), lack of knowledge (33.7 %), fear of infertility by polio vaccines (32.16 %) and security issues (29.42 %) were reported by the participants as the main barriers towards polio immunization.

**Conclusion:**

The findings of this study showed poor knowledge and negative attitudes of participants towards polio immunizations. Religious beliefs and lack of knowledge about polio immunization were reported as the major barriers towards polio immunization.

## Background

The Global Polio Eradication Initiative (GPEI) was launched in 1988 with an objective to make the world ‘polio free’. This mass campaign was a collaborative effort of national governments, World Health Organization (WHO), Centre for Disease Control and Prevention (CDC), United Nations Children’s Fund (UNICEF) and other key partners [1]. The impact of this initiative was remarkable as the number of polio cases were reduced by 99 %, from an estimated 350,000 cases in 1988 to 359 cases in 2014. However, 2 countries are still categorized as polio endemic including Pakistan and Afghanistan. Out of 359 cases reported globally in 2014, 306 cases were registered in Pakistan alone [2].

It is an alarming situation for the country as the new wave of polio has hit all the provinces of Pakistan. A sharp surge in the number of polio cases is reported from Federally Administered Tribal Areas (FATA), Khyber Pakhtunkhwa (KPK) and Balochistan. In KPK, Peshawar division is the most affected area, carrying the major burden of the disease (37 out of 68). In Balochistan, the maximum numbers of cases were reported from Quetta division (19 out of 23) [2].

Tackling polio in the last of the endemic countries has been a continuing priority for the GPEI. However, logistical barriers, especially in conflict areas; management challenges; uncertain funding; political tension; persisting anti-vaccine rumours and resistance have arrayed against the efforts of polio eradication [3]. Amidst these, public acceptance of vaccines remains important. Negative communications about polio vaccines in Nigeria resulted in decrease acceptance of vaccines in five states in Northern Nigeria. This particularly serious and well-documented set of refusals occurred due to the endorsement of rumours by religious and political leaders as they consider it as an American conspiracy to cause HIV and infertility in local population. The boycotts proved a huge setback for polio eradication in Nigeria as the number of cases jumped from 202 in 2002 to 1143 in 2006 [4]. Political motivation was another prompter of public health concern as in the case of polio vaccination boycott in Northern Nigeria, where marginalised communities were mobilized to challenge government-driven initiatives [4].

In spite of taking rigorous measures to interrupt the rapid transmission of polio virus in Pakistan [5], the success is far from reach. Security risks to healthcare workers, false religious and traditional beliefs, and limited access to hard-to-reach communities have severely hampered the progress of ‘End Polio from Pakistan’ campaign [6]. Various efforts have been made by the researchers to determine the barriers towards polio immunization in compromised settings in Pakistan [7]. Lack of knowledge and misconceptions about polio vaccination were reported as major barriers towards polio immunization by researchers from Hazara region in KPK [8]. Another study from Peshawar reported unsatisfactory knowledge of participants regarding polio immunization. However, this study was limited to urban areas of Peshawar University campus [9]. A survey conducted by Harvard Opinion Research Program in collaboration with UNICEF reported low oral polio vaccine (OPV) coverage in higher conflict areas of Pakistan (FATA), parents’ misperceptions about polio virus and OPV, and lack of parental trust on local healthcare workers [10]. Periodic evaluation of knowledge and attitudes in a population provide a basis for educational diagnosis and interventions towards changing attitudes and behaviour over time. Assessment of knowledge and attitudes of participants about polio could pave the way to design a data driven interventions to aid GPEI. The objective of this study was to examine the knowledge, attitudes and perceptions towards polio immunization among residents of Quetta and Peshawar divisions in Pakistan.

## Methods

### Study design, participants and sampling

A descriptive, cross-sectional study was conducted for the period of 5 months from August to December, 2014 in Quetta and Peshawar divisions in Pakistan. Although a large number of polio cases were reported from FATA in 2014, security threats and ban on polio vaccination from the militant group prevent the approval to include FATA in this study. The study is reported in compliance with the STROBE guidelines for observational research [11]. The study was conducted in Quetta and Peshawar divisions in view of the large number of reported polio cases in these areas in 2014 [2]. Multistage sampling technique was employed to draw a random sample from each division. First, 2 districts (out of 3) were randomly selected from Peshawar division, and 3 districts (out of 5) were randomly selected from Quetta division. Second, two tehsils were randomly selected from each of the selected district. Third, two union councils from each of the selected tehsils were randomly selected. The pre-tested questionnaire was distributed to the participants at places of common interest (shopping places, educational institutes, utility stores, restaurants etc.) throughout the respective union councils. These locations were selected for data collection in view of the availability of the socio-economically diverse population. Rural and urban areas were classified based on 1981 and 1998 population census. The same classification is used by researchers in epidemiological studies elsewhere [12, 13]. The data collectors spent an average of 5 h in each location at randomly chosen time of the day. During a 5-h period, participants were randomly approached and invited to participate in the survey. A self-administered questionnaire was used to collect the data from the participants. However, in cases where participants were unable to complete the questionnaire because of literacy issue, interviewer-assisted approach was used for data collection.

### Sample size calculation and eligibility criteria

A sample size was calculated through Raosoft software in which the power was kept as 80 %, response distribution as 50 %, while confidence interval and margin of error was set at 95 and 5 % respectively [14]. A sample size of 384 participants was generated from each division. All individuals aged 18 years and above, clinically stable, without any physical impairment were considered eligible for this study. Participants were excluded in case they were not willing to participate in this study or they were not residents of the respective locality.

### Study instrument

A questionnaire was designed after a thorough literature review of the related published studies [10, 15–20]. After drafting the first version, the questionnaire was subjected to content validity by a panel of 5 experts involving two physicians who were experts in the management of infectious diseases, one pharmacist, one epidemiologist and one sociologist. The suggested corrections were made to the questionnaire before sending them to a small sample of 20 individuals (10 from each division) for face validity. The amendments proposed by the participants in the questionnaire were incorporated in view of other published literature. The reliability coefficient of the questionnaire was calculated by using SPSS v.20. The Cronbach’s alpha value of 0.72 and 0.68 was computed for knowledge and attitude section respectively. The responses of pilot study were not included in final analysis. The English version of the questionnaire was then translated to Urdu, and then was back translated to English in accordance to the standard procedures for questionnaire translation [21, 22].

The study instrument consisted of 46 items, which were divided into four sections. The first section included 10 questions which explored the demographic information of the participants. The second section, composed of 15 questions, evaluated the knowledge of participants about the virus, sign and symptoms, risk factors, mode of transmission and immunization against polio virus. Third section examined the attitudes of respondents towards polio immunization. Participants were assessed on the basis of 8 attitudes related statements. The last section observed participants’ perceptions towards non-vaccination against polio virus. The assessment was based on participants’ responses towards 13 questions.

### Data analysis

The data were analysed by using SPSS v.20. Descriptive analysis was used to express the results as frequencies and percentages. The association between predictors and dependent variables was assessed by logistic regression analysis. The knowledge questions consisted of Yes/No response categories. Knowledge scores ranged from 0 to 15, and cut off level of <9 were set for poor knowledge and ≥9 for good knowledge. The responses of participant over attitudes statement were measured on 4 point Likert scale of agreement. A score of 1 was given to strongly disagree, 2 to disagree, 3 agree and 4 to strongly disagree. A mean score of ≥22 was considered as positive attitude while score of <22 were taken as negative attitude. A p-value of less than 0.05 was reported as statistically significant.

### Ethical approval

This study was ethically approved by research committee of Islamia College Peshawar. Moreover, the study was performed as per ethical standard for human experimentation [23]. Participation of respondents was voluntary and their responses were dealt with high level of confidentiality and anonymity. Participants were briefed about the objectives of the study and their informed consents were obtained prior to data collection.

## Results

Of 1100 individuals approached during the study period, a total of 768 participants completed the questionnaire, giving a response rate of 69.8 %. The complete information about the demographic characteristics of the participants is presented in Table 1. A total of 38.8 % participants exhibited good knowledge about polio. The results showed that participants were aware of the terminology of polio (79.1 %), and the fact that it is caused by virus (73.9 %). In contrast, 21.7 % respondents correctly answered that polio is a fatal disease. Similarly, less than one quarter (23.1 %) of respondents knew that poliomyelitis is not curable. Furthermore, a large proportion of participants (74.3 %) wrongly believed that polio vaccines should not be given to children with mild illnesses. Respondents’ knowledge about symptoms was also not very encouraging as only 29 % subjects correctly answered that most patients do not develop sub-clinical symptoms. Mean knowledge score of the participants was 7.35 ± 2.54 (Table 2).

**Table 1:** Demographic information of participants (*N*=768)

Demographic variables	Number	Percent
Age (years)		
18–30	305	39.7
31–40	175	22.8
41–50	126	16.4
51–60	111	14.4
>60	51	6.6
Gender		
Female	290	37.7
Male	478	62.2
Qualification		
Nil	150	19.5
Primary	125	16.3
Secondary	218	28.3
Religious	148	19.2
Tertiary	127	16.5
Employment		
Unemployed	258	33.6
Paid-employed	242	31.5
Self-employed	268	34.9
Income (Pakistani rupees)		
<10000	362	47.1
>40000	335	43.6
10000–40000	71	9.3
Residential Status		
Rural	333	43.4
Urban	435	56.6
Marital status		
Single	95	12.5
Married	673	87.5
Participants having children less than 5 years of age		
No	359	46.7
Yes	409	53.2
Past experience with polio patients		
No	527	68.6
Yes	241	31.4
Division		
Peshawar	384	50
Quetta	384	50

**Table 2:** Participants’ knowledge about polio

Knowledge questions	Correct answer N (%)	Incorrect answer N (%)
The terminology of ‘Poliomyelitis’	608 (79.1)	160 (20.9)
Poliomyelitis is caused by virus	568 (73.9)	200 (26.1)
The concept of infantile paralysis	495 (64.4)	273 (35.6)
Subclinical symptoms of poliomyelitis	460 (59.8)	308 (40.2)
Most persons do not develop symptoms	223 (29)	545 (71)
Lack of immunization is a risk factor	472 (61.4)	296 (38.6)
Travel to polio affected area is also a risk factor	414 (53.9)	354 (46.1)
Polio can be transmitted through direct contact from person to person	300 (39)	468 (61)
Polio can also be transmitted through from contaminated food, water and faeces	476 (61.9)	292 (38.1)
Poliomyelitis is curable	178 (23.1)	590 (76.9)
Repetition of polio vaccine	372 (48.4)	396 (51.6)
Immunization is the most effective way of preventing poliomyelitis	348 (45.3)	420 (54.7)
Polio drops should not be given to children in mild illness	198 (25.7)	570 (74.3)
Poliomyelitis can cause of death of the patient	167 (21.7)	601 (78.2)
The concept of post-polio syndrome	373 (48.5)	395 (51.4)

Note: Knowledge was assessed by giving a score of 1 to correct answer and 0 to wrong answer. The scale measured knowledge from maximum score of 15 to minimum 0. Scores < 9 were taken as poor, ≥ 9 as adequate knowledge of Polio. Mean knowledge score was 7.35 ± 2.54

The results showed that younger participants (18–30 years) had significantly higher knowledge than older (>60 years) participants (*p* < 0.001). Qualification of respondents was also significantly associated their knowledge as respondents with tertiary education were more knowledgeable than uneducated respondents (OR = 1.84, *p* < 0.05). Paid employees were more likely to have good knowledge of polio than unemployed respondents (OR = 2.11, *p* < 0.05). Residents of rural area had lower knowledge than urban population (OR = 0.51, *p* < 0.05), while residents of Quetta division were also less knowledgeable than Peshawar division (OR = 0.29, *p* < 0.001). Moreover, participants having children less than 5 years of age had higher knowledge than their respective group (OR = 2.13, *p* < 0.001). Participants who had any previous interaction with polio patient had higher knowledge than participants with no past experience with polio patient (OR = 2.20, *p* < 0.001). The associations of demographic variables with knowledge are summarized in Table 3.

**Table 3:** Association of demographic variable with the knowledge of participants towards Polio

Variables	Knowledge (%)		OR (95 % CI)	P value
	Poor	Good		
Age (years)				
18–30	36.7	63.3	Ref	
31–40	68	32	0.56 (0.34–0.93)	0.027
41–50	77	77	0.37 (0.20–0.66)	0.001
51–60	87.4	12.6	0.11 (0.05–0.23)	<0.001
>60	88.2	11.8	0.08 (0.03–0.22)	<0.001
Gender				
Female	61.4	38.6	Ref	
Male	61.1	38.9	1.26 (0.82–1.94)	0.27
Qualification				
Nil	68.7	31.3	Ref	
Primary	63.2	36.8	1.27 (0.77–2.10)	0.340
Secondary	60.1	39.9	1.45 (0.93–2.25)	0.094
Religious	59.5	40.5	1.49 (0.92–2.40)	0.098
Tertiary	54.3	45.7	1.84 (1.12–3.01)	0.015
Employment				
Unemployed	68.2	31.8	Ref	
Paid-employed	52.5	47.5	2.11 (1.19–3.76)	0.011
Self-employed	62.3	37.7	0.95 (0.57–1.59)	0.856
Income (Pakistani rupees)				
<10000	70.4	29.6	Ref	
10000–40000	53.7	46.3	2.12 (1.343.33)	0.001
>40000	49.3	50.7	2.26 (1.09–4.67)	0.028
Residential Status				
Urban	55.4	31.2	Ref	
Rural	68.8	44.6	0.51 (0.33–0.78)	0.002
Marital status				
Single	26.3	73.7	Ref	
Married	33.9	66.1	1.09 (0.4–1.18)	0.658
Participants having children less than 5 years of age				
No	63	37	Ref	
Yes	59.7	40.3	2.13 (1.37–3.30)	<0.001
Past experience with polio patients				
No	68.6	31.4	Ref	
Yes	51.9	48.1	2.20 (1.35–3.57)	<0.001
Residence				
Quetta	42.8	35.2	Ref	
Peshawar	57.2	64.8	0.29 (0.15–5.61)	<0.001

Note: Overall predictive accuracy of the model is 74.7 %

Omnibus tests of model coefficients: Chi-square value = 253.795, *p*<0.001

-2 Log Likelihood=772.030, Nagelkerke R square=0.382

Of 768 participants, 651 (84.8 %) showed negative attitudes towards polio immunization (Mean attitude score: 19.19 ± 2.39). Nearly half of participants (50.3 %) strongly agreed or agreed to the statement that all children should be vaccinated for polio. Similarly, 47.4 % participants showed negative attitudes towards appropriate storage of polio vaccines. Similarly, 58.4 % participants regarded polio as severe disease, while 63.6 % responded that the problem of polio is very severe in their area. Participants’ responses towards attitude related statements are summarized in Table 4.

**Table 4:** Participants attitudes towards Polio immunization

Attitude questions	Participants’ responses N (%)			
	Strongly disagree	Disagree	Agree	Strongly agree
Poliomyelitis is a very serious disease	130 (16.9)	189 (24.6)	392 (51)	57 (7.4)
The problem of poliomyelitis is very severe in your area	78 (10.2)	201 (26.2)	183 (23.8)	306 (39.8)
Polio vaccines are not capable to reduce the transmission of infection	65 (8.5)	246 (32)	388 (50.5)	69 (9)
Polio vaccines should be appropriately stored in order to be effective	82 (10.7)	282 (36.7)	137 (17.8)	267 (34.8)
Infected children should not be brought to public place because of risk of infection transmission	78 (10.2)	112 (14.6)	212 (27.6)	366 (47.7)
All children should be vaccinated for polio	171 (22.3)	211 (27.5)	88 (11.5)	298 (38.8)
Communities should actively participate in controlling poliomyelitis in Pakistan	120 (15.6)	235 (30.6)	114 (14.8)	299 (38.9)
People with poliomyelitis are less productive than non-disabled ones.	101 (13.2)	229 (29.8)	132 (17.2)	306 (39.8)

Note: Attitude was assessed by giving a score of 1 to strongly disagree, 2 to disagree, 3 to agree, 4 to strongly agree. The scale measured attitude from maximum score of 32 to minimum score of 8. Scores of < 22 were taken as negative attitude, and score of ≥ 22 as positive attitude. Mean attitude score was 19.19 ± 2.39

Positive attitudes of participants towards polio immunization were significantly associated with tertiary education (OR = 9.14, 0 < 0.001) and urban locality (OR = 3.29, *p* < 0.001). Respondents having children less than 5 years of age showed positive attitudes towards polio vaccination than their respective counterparts (OR = 2.49. *p* < 0.05). The results showed that participants with religious qualification had positive attitudes towards polio immunization than participants with no formal qualification (OR = 5.89, *p* < 0.001). Similarly, paid employees had negative attitudes towards polio immunization than unemployed participants (OR = 0.10, *p* < 0.001). The associations of demographic variables with attitudes are tabularized in Table 5. Religious beliefs (39.06 %), lack of knowledge (33.7 %), fear of infertility by polio vaccines (32.16 %) and security issues (29.42 %) were the main barriers towards polio immunization (Table 6).

**Table 5:** Association of demographic variable with the attitudes of participants towards Polio immunization

Variables	Attitudes (%)		OR (95 % CI)	P value
	Negative	Positive		
Age (years)				
18–30	83	17	Ref	
31–40	82.3	17.7	2.34 (1.1–4.8)	0.021
41–50	91.3	8.7	0.33 (0.14–0.78)	0.012
51–60	88.3	11.7	0.87 (0.38–1.98)	0.752
>60	80.4	19.6	1.50 (0.57–3.93)	0.405
Gender				
Female	85.5	14.5	Ref	
Male	84.3	15.7	0.61 (0.34–1.08)	0.094
Qualification				
Nil	94	6	Ref	
Primary	76.8	23.2	3.03 (1.14–8.0)	0.025
Secondary	91.3	8.7	2.01 (0.76–5.31)	0.157
Religious	79.1	20.9	5.89 (2.17–15.9)	<0.001
Tertiary	77.2	22.8	9.14 (3.39–24.6)	<0.001
Employment				
Unemployed	83.7	16.3	Ref	
Paid-employed	94.2	5.8	0.10 (0.04–0.23)	<0.001
Self-employed	77.2	22.8	1.29 (0.72–2.31)	0.390
Income (Pakistani rupees)				
<10000	88.4	11.6	Ref	
10000–40000	80.6	19.4	3.55 (1.34–9.41)	0.002
>40000	85.9	14.1	2.67 (1.44–4.94)	0.011
Residential Status				
Rural	77.2	22.8	Ref	
Urban	90.6	9.4	3.29 (1.87–5.79)	<0.001
Marital status				
Single	73.7	26.3	Ref	
Married	86.3	13.7	0.71 (0.29–1.69)	0.441
Participants having children less than 5 years of age				
No	89	11	Ref	
Yes	79.9	20.1	2.49 (1.28–4.87)	0.015
Past experience with polio patients				
No	82.4	17.6	Ref	
Yes	87.7	12.3	0.53 (0.27–1.01)	0.057
Residence				
Peshawar	86.2	13.8	Ref	
Quetta	83.1	16.9	1.38 (0.65–2.91)	0.396

Note: Overall predictive accuracy of the model is 87.4 %

Omnibus tests of model coefficients: Chi-square value = 152.11, *p*<0.001

-2 Log Likelihood= 503.375, Nagelkerke R square= 0.313

**Table 6:** Barriers towards polio immunization

Reasons	N	%
Lack of knowledge	259	33.7
Lack of time / Busy schedule	87	11.32
Non- compliant spouse	89	11.58
Religious belief	300	39.06
Misconceptions about vaccination	166	21.61
Lack of immunization services	120	15.62
Vaccination is considered harmful	190	24.73
Lack of trust on healthcare workers	190	24.73
Lack of trust on the quality of vaccines	168	21.87
Fear of side effects	189	24.60
Polio vaccines causes infertility etc.	247	32.16
Vaccination is not considered necessary	31	4.03
Security issues	226	29.42
Others	6	0.78 %

Note: % values may not add up to 100 since respondents may have selected more than one reason

## Discussion

The results showed that participants were aware of the terminology of polio and its existence as a disease. Although these results were comparatively better than the other knowledge questions, the lack of understanding of polio in remaining 20.9 % participants may have far reaching implications. These results are balanced in view of other existing literature [24, 25]. However, the results are relatively better than a study conducted in Karachi, Pakistan, where 55.4 % participants knew about polio [20]. The probable reason of this discrepancy could be due to intensification of polio campaigns in past few months in Pakistan as the referenced study was conducted in late 2011. The overall knowledge of participants about polio was inadequate (38.8 %) in view of other published study [26]. Efforts have been made by the non-governmental organisations (NGOs) such as UNICEF to expand the social mobilization programs in polio affected areas by engaging community leaders, civil society organizations, and community members to ensure that polio immunization is recognized as an essential practice to protect the health of all children [20]. The utilization of mass media campaigns and information dissemination approaches along with the planned communication strategies such as social mobilization could be valuable to address the challenges faced in the remote areas of Pakistan.

It is noteworthy to mention that our study highlighted the significant association of knowledge with the age of the participants. Youngsters appeared to be more knowledgeable than older ones. The emergence of social media websites as popular health information for young adults could be the likely reason of this finding [27]. Further studies are warranted to validate these results, and interventions should be customized accordingly. The point of concern here is that the older person in a family is also a decision maker in Pakistani society. This person has the authority to take decision relating to different aspects of life including whether or not to vaccinate children in a family. Our findings emphasize on the need to educate the head of a family as their knowledge and positive attitudes towards vaccination are essential for polio eradication. Participants with low income, no formal education and residents of rural locality are also less likely to be knowledgeable about polio. These findings are in line with other reported studies [28–30]. Social depravity and low accessibility by healthcare workers mainly reflects such findings. This opinion is also supported by other researchers who reported higher immunization rate among people who had healthcare centre and/or vaccination facility within 12 km of their residence [31].

A substantial proportion of participants displayed negative attitudes towards polio immunization. Public attitudes towards most of the attitude related statements were negative. However, it is worth discussing the negative attitudes of respondents with regards to storage of polio vaccines. Polio vaccines require cold chain maintenance that should begin from the manufacturing site till the administration of vaccines to the patient. A slightest of change in temperature may alter the molecular structure of the vaccines, which in turn may render them ineffective [19]. Many researchers have emphasized on the need to enhance community awareness of the cold chain maintenance of polio vaccines in Pakistan [17]. Necessary measures should be taken to design comprehensive educational programs for the public to educate them regarding all major components of the cold chain system. Moreover, severe electrical load shedding (power cuts) in Pakistan is also a hurdle in cold chain maintenance of polio vaccines. It is required to take into consideration these factors to devise strategies to counter such problems. Efforts should also be made to target rural areas where the negative attitudes towards polio vaccination are more prevalent. These efforts could include the identification and subsequent counselling of the leaders/decision makers of rural areas, as those people are viewed as highly respectful individuals. This strategy could prove to be effective in shaping the attitudes of the rural residents in timely manner. Special reforms are required to improve attitudes of the people along with their knowledge of polio to aid in turning the dream of polio-free world into reality for all the children.

Religious and social beliefs appeared to be the major barrier preventing the disease from tipping over into complete eradication. The local religious extremists in the tribal areas of Pakistan have denounced polio vaccination as an American ploy to sterilize Muslim populations [18]. Similar reasons are also reported by researchers in other published studies [18, 20, 26–30]. Moreover, immunization drives have also become a victim of political instability in Pakistan as the polio vaccination campaigns have proved a popular target for those opposing the government and foreign policy. To interpret the findings about religious beliefs, we need to understand the basic principles of Islam on this issue. Trypsin, a component of oral polio vaccine, is sometimes extracted from pork pancreas, which is considered as *haram* (prohibited) in Islam. However, trypsin is used in minute quantity in the vaccine, and according to the principles of Islam, when the quantity of water exceeds 2 *qilla,* the effect of impurities is negligible [32]. The Grand Mufti (Supreme Muslim Cleric) of Saudi Arabia has also supported the cause of Polio-free world. We believe that coalition campaign involving Imams, Sheikhs and other Muslim scholars could turn the tide against polio vaccines in Pakistan. This suggestion is also supported by researchers elsewhere [20].

We believe that our findings are important from global perspective, especially other polio endemic country like Afghanistan. Poor knowledge and false religious beliefs are important drivers in the way people understand the disease. These factors are very likely to be the source of misconception and potential barriers to behaviours change. Based on our findings, and other reported literature [33], it would be safe to say that the transfer of knowledge from trusted sources like community leaders and religious scholars could significantly reduce the burden of polio from the affected countries. We suggest that the whole campaign should be divided into 2 domains; one should be responsible for the education and the counselling of the people, while the other domain can focus on vaccination and reaching out remote areas which are still not accessed. We would encourage the researchers to conduct studies to assess how individuals’ beliefs and attitudes changes over time, the determinants of their vaccination decision, and the sources of information which strongly influences their decision. Additionally, there is also a need to evaluate the effectiveness of communication strategies between the healthcare providers and the parents.

The strength of this study is that it has focused on the area where the availability of literature is limited. Inclusion of participants from areas highly affected by polio is another key feature of this study. The results of this study would help the stakeholders and other health officials to evaluate the effectiveness of their policies about the eradication of polio from Pakistan. However, cautions should be taken while interpreting these results as they may not be generalizable to the whole country. The findings of this study represent Quetta and Peshawar divisions of Pakistan which may not account for the broader Pakistani population. Since the participation in this study was voluntary, we cannot ignore the potential for self-selection bias by community members who are more concerned about polio. The participants were approached randomly to collect data, hence they may not account for the differences within the population. Although it does not reflect internal validity of the findings, it may decrease the overall generalizability of the findings. No attempt was made to study the direct correlation between the findings of this study and the high incidence of polio in study areas. As a general limitation to self-administered and interviewer-assisted questionnaire, we cannot ignore the tendency of participants to provide more socially desirable responses.

## Conclusion

The study findings showed poor knowledge and negative attitudes of participants towards polio immunization. Religious belief was the major barrier towards polio immunization. Special attention should be given to educate people about polio to enhance their acceptability of polio vaccines. Interventions should be customized to target participants more likely to be associated with poor knowledge and negative attitudes towards polio immunization.
